# Biofilm-Forming *Staphylococcus epidermidis* Expressing Vancomycin Resistance Early after Adhesion to a Metal Surface

**DOI:** 10.1155/2015/943056

**Published:** 2015-01-31

**Authors:** Toshiyuki Sakimura, Shiro Kajiyama, Shinji Adachi, Ko Chiba, Akihiko Yonekura, Masato Tomita, Hironobu Koseki, Takashi Miyamoto, Toshiyuki Tsurumoto, Makoto Osaki

**Affiliations:** ^1^Department of Orthopaedic Surgery, Graduate School of Biomedical Sciences, Nagasaki University, Nagasaki 852-8501, Japan; ^2^Department of Gross Anatomy, Graduate School of Biomedical Sciences, Nagasaki University, Nagasaki 852-8501, Japan

## Abstract

We investigated biofilm formation and time of vancomycin (VCM) resistance expression after adhesion to a metal surface in *Staphylococcus epidermidis*. Biofilm-forming *Staphylococcus epidermidis* with a VCM MIC of 1 *μ*g/mL was used. The bacteria were made to adhere to a stainless steel washer and treated with VCM at different times and concentrations. VCM was administered 0, 2, 4, and 8 hours after adhesion. The amount of biofilm formed was evaluated based on the biofilm coverage rates (BCRs) before and after VCM administration, bacterial viability in biofilm was visually observed using the fluorescence staining method, and the viable bacterial count in biofilm was measured. The VCM concentration required to decrease BCR significantly compared with that of VCM-untreated bacteria was 4 *μ*g/mL, even in the 0 hr group. In the 4 and 8 hr groups, VCM could not inhibit biofilm growth even at 1,024 *μ*g/mL. In the 8 hr group, viable bacteria remained in biofilm at a count of 10^4^ CFU even at a high VCM concentration (1,024 *μ*g/mL). It was suggested that biofilm-forming *Staphylococcus epidermidis* expresses resistance to VCM early after adhesion to a metal surface. Resistance increased over time after adhesion as the biofilm formed, and strong resistance was expressed 4–8 hours after adhesion.

## 1. Introduction

Biomaterials are used in a variety of medical fields and have contributed to medical development. In the orthopedic field, metal implants are used in many types of surgery as materials for osteosynthesis for fracture, artificial joints for articular diseases, and fixation for spinal surgery and exhibit marked therapeutic effects. However, infection around implants is a serious complication of surgery using implants and is known as implant-related infection. Implant-related infection is intractable, and, once it occurs, long-term treatment may be necessary, including several surgeries; implant removal is also inevitable in some cases [1, 2].

Biofilm formed by bacteria is involved in the intractability of implant-related infection [3–5]. When bacteria adhere to the implant surface and grow, extracellular matrix, called extracellular polysaccharides (EPSs), is produced over time and surrounds the bacterial cells. The structure wrapped by polysaccharide-containing matrix produced by bacteria is called biofilm. Once bacteria adhere to the implant surface and form biofilm, they show behavior that differs from that of floating bacteria, become resistant to treatment including antibiotic treatment, and change immune function [1, 6–8].

*Staphylococcus* species account for more than half of the causative bacteria of cases of implant-related infection [1, 9–11].* Staphylococcus epidermidis* has strong implant-adhering ability, and its biofilm-forming ability is considered as a serious pathogenic factor [12–14]. Generally, antibiotics including cephems are considered effective for staphylococcal infections, and a glycopeptide, vancomycin (VCM), is a potent antibiotic exhibiting a bactericidal effect on most gram-positive bacteria including methicillin-resistant* Staphylococcus aureus* (MRSA) [15–17]. However, bacteria that form biofilm show strong resistance to various antibiotics, and the low sensitivity to VCM of bacteria that form biofilm has been reported [18–20].

Many findings concerning the strong drug resistance of bacteria that form biofilm have been obtained. Ceri et al. reported that the minimum biofilm eradication concentration (MBEC) was far higher than the minimum growth-inhibitory concentration (MIC) on evaluation using the Calgary Biofilm Device [21], and Pettit et al. reported that the minimum biofilm growth-inhibitory concentration (MBIC) was higher than the MIC on alamarBlue assay [22]. Nishimura et al. investigated staphylococcal biofilm formation detected in infected joint replacement cases using the microtiter plate method and observed that bacteria that formed biofilm were 1,000 times or more resistant to antibiotics than floating bacteria [23]. Biofilm formed by bacteria may thus markedly aggravate implant-related infection, and the establishment of an appropriate treatment method is awaited. Antibiotic administration in the early phase in which infection is established may be effective, but it is unclear when implant-adhering bacteria express resistance to antibiotics in the biofilm formation process. The objective of this study was to investigate the time of drug resistance expression with biofilm growth in metal surface-adhering bacteria.

## 2. Materials and Methods

### 2.1. Bacterial Species, Antibiotic, and Metal for Bacterial Adhesion

*Staphylococcus epidermidis* is a typical etiologic agent of implant-related infection. The standard biofilm-forming strain, RP62A (ATCC35984), was used as the test bacteria. For the antibiotic, VCM (vancomycin hydrochloride; Wako, Osaka, Japan), to which the bacteria are sensitive, was used. The MIC of VCM for the test bacteria was 1 *μ*g/mL on a test performed by the broth microdilution method beforehand. For the metal material to be adhered to by the bacteria, a stainless steel washer with 6.0 mm diameter and 0.5 mm thickness (UW-0306-05, Wilco, Tokyo, Japan) was used after ultrasonic cleaning and sterilization using an autoclave.

### 2.2. Preparation of Bacterial Suspension, Biofilm Formation, and VCM Administration

The test bacteria were preliminarily cultured overnight at 37°C in liquid medium, trypticase soy broth (TSB) (Becton-Dickinson, Sparks, MD, USA). The culture was diluted 10 times with fresh TSB and further grown at 37°C to a concentration of OD_600_ = 0.2 (2.0 × 10^7^ CFU/mL) using the growth curve prepared in a preliminary experiment as a reference, and a suspension of bacteria in the logarithmic growth phase was prepared. To a 24-well polystyrene plate containing washers (24-well microplate; Iwaki, Funabashi, Japan), 1 mL of the bacterial suspension was added to each well and the bacteria were allowed to adhere to the washer for 5 minutes. After washing twice with phosphate-buffered saline (PBS) to eliminate floating bacteria, the washers with adherent bacteria were incubated in 1 mL of fresh TSB for 0, 2, 4, and 8 hours to allow the bacteria to form biofilm on the washer surface, being designated as 0 hr, 2 hr, 4 hr, and 8 hr groups, respectively. The washers of the 4 groups with biofilm formation were washed twice with PBS. Each washer was transferred into 1 mL of TSB containing 0–1,024 *μ*g/mL VCM to start VCM treatment, and the following evaluation was carried out after 20-hour culture at 37°C.

### 2.3. Biofilm Coverage Rate (BCR)

The BCR on the washer was calculated following the method reported by Kajiyama et al. [24]. The washer was fixed with 95% ethanol for 1 minute, dried, and stained with 0.5% crystal violet for 5 minutes, followed by washing with distilled water. After drying, the washer was observed under a digital stereoscopic microscope (VHX-100; Keyence, Osaka, Japan). Eight regions were randomly selected in each washer and imaged at 450-times magnification. BCR was calculated from the images using image analysis software, Image J (National Institutes of Health, USA). The experiment was repeated 5 times, and BCR was calculated at 80 sites for each condition.

### 2.4. Fluorescence Staining Method

For visual observation of the distribution of live and dead bacteria in biofilm, LIVE/DEAD* Bac*Light (Invitrogen Molecular Probes, Eugene, OR, USA) was used. SYTO9, which stains live bacteria with green fluorescence, and propidium iodide, which stains dead bacteria with red fluorescence, were mixed at 1 : 1 and combined with PBS by adding 3 *μ*L of the mixture per 1 mL of PBS to prepare a stain solution. The washers were placed in the stain solution and reacted for 15 minutes. The stained washers were observed under a fluorescence microscope (BZ-8100; Keyence, Osaka, Japan), and the distribution of green live and red dead bacteria was evaluated.

### 2.5. Viable Cell Count (VCC)

The VCC in biofilm was measured following the method reported by Kajiyama et al. [24]. The washer was placed in a 1.5 mL microtube containing 500 *μ*L of PBS, shaken for 1 minute, sonicated for 3 minutes, and shaken for 1 minute to detach bacteria in biofilm from the washer. The suspension in the microtube was combined with an additional 500 *μ*L of PBS, mixed, and inoculated on medium for bacterial count measurement, Compact Dry Nissui TC (Nissui Pharmaceutical, Tokyo, Japan), using the plate dilution method, and the VCC was measured. The experiment was repeated 5 times.

### 2.6. Statistical Analysis

The results of BCR and VCC were subjected to two-way ANOVA and multiple comparison using the Bonferroni method, and *P* < 0.05 was regarded as significant. All statistical analyses were performed using SPSS version 22.0 for Windows (IBM).

## 3. Results

### 3.1. BCR and VCC after Adhesion

**Figure 1:**
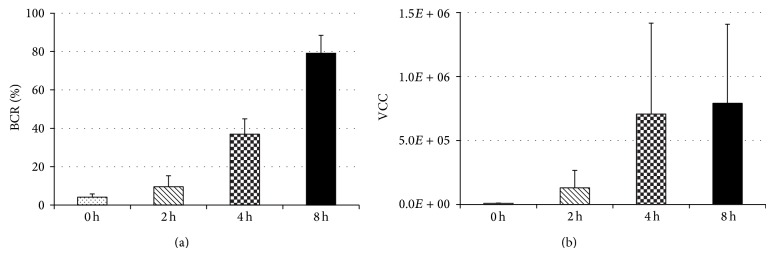
BCR and VCC after adhesion. After 5-minute adhesion to the washer surface, the bacteria were cultured and formed biofilm in TSB for 0, 2, 4, and 8 hours. BCRs on the washer surface were 4.1, 9.5, 37.0, and 79.2% in the immediate, 2 hr, 4 hr, and 8 hr groups, respectively, showing a significant increase with prolongation of the culture time in TSB (a). VCCs in biofilm were 7.2 × 10^3^, 1.3 × 10^5^, 7.0 × 10^5^, and 7.9 × 10^5^ CFU, respectively, showing no significant difference among the groups (b). Values represent mean and error bars indicate SD (*n* = 80 (a), *n* = 5 (b)).

When the bacteria adhered to the washer surface for 5 minutes, followed by culture in TSB for 0, 2, 4, and 8 hours for biofilm formation, BCRs on the washer surface were 4.1, 9.5, 37.0, and 79.2% in the 0 hr, 2 hr, 4 hr, and 8 hr groups, respectively, showing that BCR significantly increased with culture time in TSB (Figure 1(a)). VCCs in biofilm were 7.2 × 10^3^, 1.3 × 10^5^, 7.0 × 10^5^, and 7.9 × 10^5^ CFU, respectively, but no significant difference was noted among the groups (Figure 1(b)).

### 3.2. Changes in BCR with Changes in the VCM Concentration Administered at Various Time Points of Biofilm Formation

**Figure 2:**
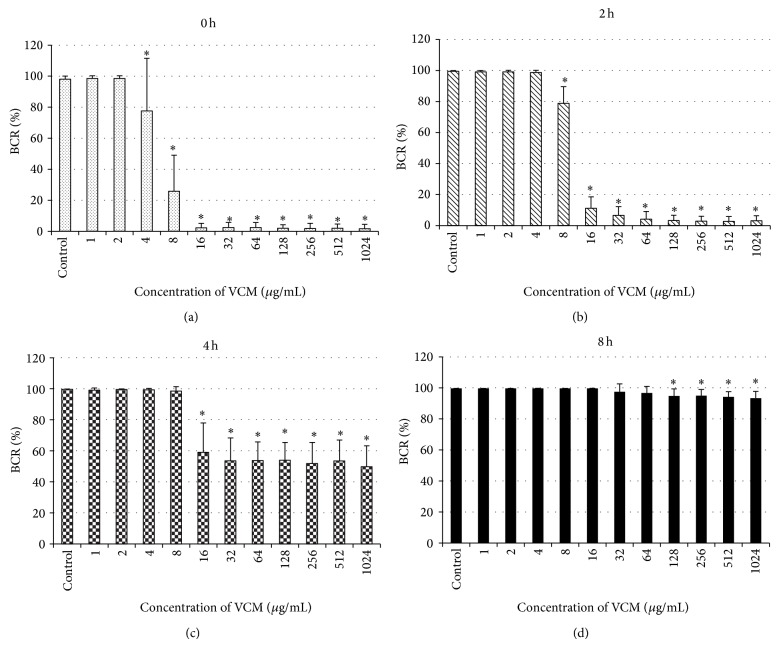
Changes in BCR with changes in the VCM concentration administered at various time points of biofilm formation. In the immediate group, BCR after 20-hour culture was 98% or higher when the VCM concentration was 2 *μ*g/mL or lower (a). BCR significantly decreased when the VCM concentration was 4 *μ*g/mL or higher(a). In the 2 hr group, BCR was 98% or higher when the VCM concentration was 4 *μ*g/mL or lower. BCR significantly decreased to 78.8% at 8 *μ*g/mL (b). In the 4 hr group, BCR was 98% or higher when the VCM concentration was 8 *μ*g/mL or lower, it significantly decreased to 59.1% at 16 *μ*g/mL, and it was mostly constant at 32 *μ*g/mL or higher (53.9–49.8%) (c). In the 8 hr group, BCR was 96% or higher when the VCM concentration was 64 *μ*g/mL or lower, it significantly decreased to 94.8% at 128 *μ*g/mL, and it was mostly constant at 256 *μ*g/mL or higher (94.9–93.4%) (d). Values represent mean and error bars indicate SD (*n* = 80). ^*^ *P* < 0.001 versus control.

Changes in BCR after 20-hour culture in the presence of VCM at various concentrations are shown in Figure 2. In the 0 hr group, BCR of the control was 98.0%, and BCR after 20-hour culture in the presence of VCM at 2 *μ*g/mL or lower was 98% or higher (Figure 2(a)). BCR significantly decreased when the VCM concentration was 4 *μ*g/mL or higher, and those at 16 *μ*g/mL or higher VCM were lower than that (4.1%) before VCM treatment (Figure 2(a)). In the 2 hr group treated with VCM after biofilm formation for 2 hours after adhesion, BCR of the control was 99.5%, and BCR was 98% or higher when the VCM concentration was 4 *μ*g/mL or lower. BCR significantly decreased to 78.8, 11.2, and 6.5% when the VCM concentrations were 8, 16, and 32 *μ*g/mL, respectively (Figure 2(b)). When the VCM concentration was 64 *μ*g/mL or higher, BCR was lower (Figure 2(b)) than that (9.5%) before VCM administration (Figure 1(a)). In the 4 hr group treated with VCM after 4-hour biofilm formation, BCR of the control was 99.6%, and BCR was 98% or higher when the VCM concentration was 8 *μ*g/mL or lower. It significantly decreased to 59.1% when the VCM concentration was 16 *μ*g/mL, but it was higher than that (37.0%) before VCM treatment when the VCM concentration was 32 *μ*g/mL or higher (Figure 1(a)), and BCR was mostly constant (53.9–49.8%) (Figure 2(c)). In the 8 hr group treated with VCM after 8-hour biofilm formation, BCR of the control was 99.7%, and BCR was 96% or higher at 64 *μ*g/mL or lower VCM concentration, and it significantly decreased to 94.8% at 128 *μ*g/mL but did not become lower than that (79.2%) before VCM administration (Figure 1(a)), even when the VCM concentration was 256 *μ*g/mL or higher, and BCR was almost constant (94.9–93.4%) (Figure 2(d)).

### 3.3. Changes in the Distribution of Live and Dead Bacteria with Changes in the VCM Concentration Administered at Various Time Points of Biofilm Formation by Fluorescence Staining Method

**Figure 3:**
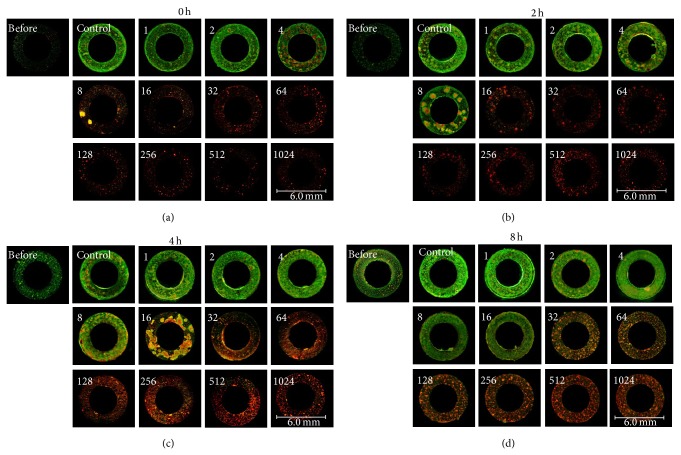
Changes in the distribution of live and dead bacteria with changes in the VCM concentration administered at various time points of biofilm formation on fluorescence staining using LIVE/DEAD* Bac*Light. The cultures were observed under a fluorescence microscope (8x magnification). Numbers in the figures represent the VCM concentrations (*μ*g/mL). In the immediate group, most bacteria were alive, stained green, when the VCM concentration was 2 *μ*g/mL or lower, similarly to those in the control group. Dead bacteria, stained red, surrounded by live bacteria appeared as spots at 4 *μ*g/mL, and the rate of live bacteria decreased and the rate of dead bacteria increased at 8 *μ*g/mL. Most bacteria were dead at 16 *μ*g/mL or higher (a). In the 2 hr group, most bacteria were alive when the VCM concentration was 4 *μ*g/mL or lower. Dead bacteria surrounded by live bacteria appeared as spots at 8 *μ*g/mL, and the rate of live bacteria decreased and the rate of dead bacteria increased at 16 *μ*g/mL. Most bacteria were dead at 32 *μ*g/mL or higher (b). In the 4 hr group, most bacteria were alive when the VCM concentration was 4 *μ*g/mL or lower. Dead bacteria surrounded by live bacteria appeared as spots at 8 *μ*g/mL, these spots of dead bacteria expanded at 16 *μ*g/mL, and the rate of live bacteria decreased at 32 *μ*g/mL. Most bacteria were dead at 62 *μ*g/mL or higher (c). In the 8 hr group, most bacteria were alive when the VCM concentration was 16 *μ*g/mL or lower. The rate of live bacteria decreased and the rate of dead bacteria increased at 32 *μ*g/mL, and most bacteria were dead at 64 *μ*g/mL or higher (d).

Changes in the distribution of live and dead bacteria were investigated by fluorescence staining using LIVE/DEAD* Bac*Light (Figure 3). In the 0 hr group treated with VCM immediately after adhesion and cultured for 20 hours, most cells were stained green, representing viable bacteria, similar to the control, when the VCM concentration was 2 *μ*g/mL or lower. At 4 *μ*g/mL, red spots representing dead bacteria, apparently surrounded by live bacteria, became conspicuous. At 8 *μ*g/mL, the rate of live bacteria decreased with an increase in the rate of dead bacteria. At 16 *μ*g/mL or higher, most cells were dead (Figure 3(a)). In the 2 hr group treated with VCM after 2-hour biofilm formation, most bacteria were alive at a VCM concentration of 4 *μ*g/mL or lower. Spots of dead bacteria, apparently surrounded by live bacteria, became conspicuous at 8 *μ*g/mL, and the rate of live bacteria decreased with an increase in the rate of dead bacteria at 16 *μ*g/mL. At 32 *μ*g/mL or higher, most cells were dead (Figure 3(b)). In the 4 hr group that formed biofilm for 4 hours, most cells were alive at a VCM concentration of 4 *μ*g/mL or lower. Dead bacteria surrounded by live bacteria were conspicuous as spots at 8 *μ*g/mL, these spots of dead bacteria increased at 16 *μ*g/mL, and the rate of live bacteria decreased and the rate of dead bacteria increased at 32 *μ*g/mL. Most cells were dead at 62 *μ*g/mL or higher (Figure 3(c)). In the 8 hr group that formed biofilm for 8 hours, most cells were alive at 16 *μ*g/mL or lower. The rate of live bacteria decreased and the rate of dead bacteria increased at 32 *μ*g/mL. Most cells were dead at 64 *μ*g/mL or higher (Figure 3(d)).

### 3.4. Changes in VCC with Changes in the VCM Concentration Administered at Various Time Points of Biofilm Formation

**Figure 4:**
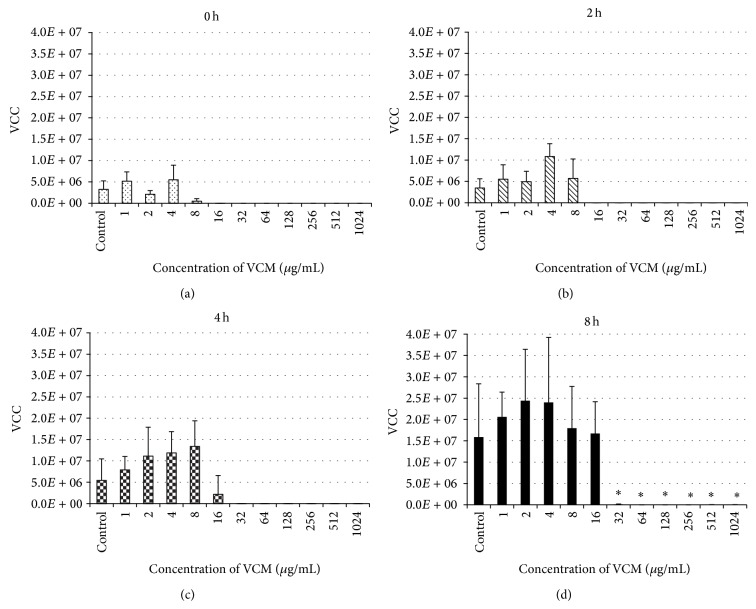
Changes in VCC with changes in the VCM concentration administered at various time points of biofilm formation. No significant changes were noted in the viable cell count in biofilm after 20-hour culture among the VCM concentrations in the immediate, 2 hr, or 4 hr group ((a), (b), (c)). VCC was 10^7^–10^5^ CFU when the VCM concentration was 0–8 *μ*g/mL in the immediate, 2 hr, and 4 hr groups. The count was only 10^3^ CFU at 16 *μ*g/mL and 0–10 CFU at 32 *μ*g/mL or higher in the immediate and 2 hr groups. In the 4 hr group, the count was 10^2^ CFU at 32 *μ*g/mL or higher and only 10 CFU at 64 *μ*g/mL or higher. In the 8 hr group, the count was 10^7^–10^5^ CFU after 20-hour culture in the presence of VCM at 16 *μ*g/mL or lower, showing no effect of VCM, and it significantly decreased at 32 *μ*g/mL or higher (d), but the count was still high (2.4 × 10^4^ CFU) even at the highest VCM concentration (1,024 *μ*g/mL). Values represent mean and error bars indicate SD (*n* = 5). ^*^ *P* < 0.001 versus control.

In the 0, 2, and 4 hr groups, the graph shows increases in the VCC with delay of the beginning of administration at all VCM concentrations, but no significant difference was observed in the VCC in biofilm after 20-hour culture among the groups (Figures 4(a), 4(b), and 4(c)). In the 0, 2, and 4 hr groups, the VCC was 10^7^–10^5^ CFU at VCM concentrations of 0–8 *μ*g/mL. In the 0 hr and 2 hr groups, the count was only 10^3^ CFU at 16 *μ*g/mL and 0–10 CFU at 32 *μ*g/mL or higher. In the 4 hr group, the count was 10^2^ CFU at 32 *μ*g/mL or higher and only 10 CFU at 64 *μ*g/mL or higher. In the 8 hr group, the count after 20 hour culture with VCM was 10^7^–10^5^ CFU at 16 *μ*g/mL or lower VCM, showing no effect of VCM, and the count significantly decreased at 32 *μ*g/mL or higher (Figure 4(d)), but it was still high, 2.4 × 10^4^ CFU, even in the presence of VCM at the highest concentration (1,024 *μ*g/mL).

## 4. Discussion

Although the process of antibiotic resistance expression in biofilm formation in adherent bacteria was investigated in many studies, many points remain unclear. We investigated the time of VCM resistance expression of* Staphylococcus epidermidis*. When changes in the amount of biofilm formed were investigated, although the MIC of VCM for the test bacteria was 1 *μ*g/mL, a concentration of 4 *μ*g/mL or higher was necessary to reduce biofilm formation significantly when the bacteria were treated with VCM immediately after adhesion (Figure 2(a)). Moreover, complete inhibition of biofilm growth without an increase in BCR from that at treatment initiation could not be achieved even though VCM was administered immediately after adhesion, unless the VCM concentration was increased to 16 *μ*g/mL or higher, clarifying that* Staphylococcus epidermidis* expresses antibiotic resistance immediately after adhesion to metal. Miyake et al. performed a study with* Staphylococcus aureus* adhering to a plastic tissue culture plate, in which the bacteria already showed antibiotic resistance to some extent before biofilm formation [25], which does not contradict our findings. It is also considered that the expression of many genes and proteins is involved in the mechanism of antibiotic resistance expression of bacteria based on previous studies [26–29]. Since bacteria expressed antibiotic resistance immediately after adhesion, it is assumed that bacterial adhesion triggers the expression of certain genes and proteins and these are involved in VCM resistance, in addition to the extracellular matrix acting as a barrier.

There is no appropriate index of the strength of antibiotic resistance of biofilm, and its investigation is necessary. We investigated the VCM concentration inhibiting BCR elevation as an index of VCM resistance. The results indicated increases in the resistance of* Staphylococcus epidermidis* to VCM with biofilm formation. Moreover, when VCM was administered after allowing biofilm to develop for 4 hours or longer, biofilm formation could not be inhibited completely even at a high VCM concentration. Resistance to VCM increased as biofilm is formed with a delay of the beginning of VCM administration.

The distribution of the antibiotic action in biofilm was investigated using LIVE/DEAD* Bac*Light. As a result, the concentration at which the color indicating live bacteria changed to that indicating dead bacteria was in agreement with the concentration at which the VCC decreased to 10^5^ CFU or less in all time-based groups. In addition, in a study using LIVE/DEAD* Bac*Light, multiple spots considered to represent microcolonies became conspicuous at half the VCM concentration that caused color change, and close examination of each spot confirmed that dead bacteria were surrounded by live ones. This phenomenon may have been due to the accumulation of bacteria killed by VCM in the center of microcolonies. This finding is consistent with those in the study reported by Singh et al. [30], in which the spatial distribution of VCM-induced damage of* Staphylococcus epidermidis* biofilm was revealed by electron microscopy. They clarified that the effect of VCM differed between the center and periphery of* Staphylococcus epidermidis* biofilm, and the central region was damaged by VCM, whereas VCM was ineffective for the periphery. Similarly, the VCM resistance of the bacteria present in the marginal region of biofilm may have been stronger than that in the central region in our study.

The influences of VCM administered at different time points of biofilm formation on changes in the amount of biofilm formed and the distribution of live and dead bacteria were clarified, but changes in the viable bacterial count in biofilm were unclear. When the changes in the count after VCM administration were investigated, live bacteria decreased to a very small number in the presence of 16 *μ*g/mL or higher VCM in the groups treated with VCM within 2 hours after adhesion and 32 *μ*g/mL or higher in the group treated after 4-hour biofilm formation, but a high count of live bacteria (10^4^ CFU or higher) was detected even at a high VCM concentration (32–1,024 *μ*g/mL) in the group treated after 8-hour biofilm formation (Figure 3(d)). VCM exhibited a bactericidal effect when it was administered at a high concentration within 4 hours after adhesion, but relatively many bacteria survived in biofilm even though VCM was present at a high concentration when 8 hours had passed after adhesion, suggesting that* Staphylococcus epidermidis* adhering to metal acquires strong resistance to antibiotics 4–8 hours after adhesion. Kajiyama et al. reported that the rate of increase in BCR at 5-6 hours after adhesion was significantly high [24], which was consistent with the time of expression of strong antibiotic resistance in our study.

Generally, drug sensitivity is evaluated based on tests with floating bacteria, but the test results should be interpreted carefully because drug resistance increases in bacteria adhering to solid surfaces. It was clarified that, unlike floating bacteria, bacteria adhering to metal expressed resistance to antibiotics early after adhesion, and strong resistance was noted 8 hours after adhesion. Antibiotic resistance may differ markedly between floating bacteria and bacteria that form biofilms in clinical cases, which may be problematic. Currently, determination of MIC using the broth microdilution method is mainly employed for drug sensitivity tests to select antibiotics, but floating bacteria are tested by this method, and the sensitivity of bacteria adhering to solid surfaces or present in biofilm is not investigated. For the treatment of implant-related infection, antibiotics should not be selected based on the MIC value alone, and it may be better to consider the use of antibiotics with an antibiofilm effect early after onset in consideration of the antibiotic resistance of bacteria adhering to the implant. Minocycline, daptomycin, tigecycline, and rifampicin have been reported to be effective against bacteria in biofilm [2, 18, 19, 31]. The use of these antibiotics should be investigated. According to the results of this study, early and high-dose administration of an antibiotic is considered to be effective for controlling bacteria adhering to implants. Clinically, however, it is important to devise a treatment plan in consideration of the characteristics of the pharmacokinetics and antibacterial activity (pharmacodynamics) of the drug to be used. Characteristics of the antibiotic such as whether its activity is concentration-dependent or time-dependent must be taken into consideration, and a dosing schedule that provides the maximum effect within the safe blood concentration range must be developed through plasma drug concentration monitoring. In addition, as the results of this study indicate, bacteria exhibit strong drug resistance once they form biofilm. Therefore, as the literature suggests, it is also necessary to recognize the importance of prophylactic antibiotic therapy for the early use of antibiotics, possibly before or shortly after the adhesion of bacteria to implants.

As a limitation of this study, the experiments were performed using only the standard biofilm-forming strain of* Staphylococcus epidermidis*. It remains to be investigated whether or not similar results can be obtained from other strains of* Staphylococcus epidermidis* and clinical isolates. BCR used to evaluate the amount of biofilm formed is simple and reproducible, but it is a 2-dimensional method and may not be applicable for long-term observation after biofilm formation because biofilm grows 3-dimensionally. However, it is not problematic for evaluation of a relatively early phase after adhesion. The evaluation of other types and combinations of antibiotics and effects of metal types and surface processing is possible using this experimental system, and the acquisition of new information from these is expected.

In conclusion, it was suggested that biofilm-forming* Staphylococcus epidermidis* adhering to a metal surface expresses VCM resistance early after adhesion. VCM resistance increased as initiation of VCM treatment was delayed and biofilm was formed, and strong antibiotic resistance may have been expressed 4–8 hours after adhesion.
